# Annual 30-m big Lake Maps of the Tibetan Plateau in 1991–2018

**DOI:** 10.1038/s41597-022-01275-9

**Published:** 2022-04-12

**Authors:** Rui Zhao, Ping Fu, Yan Zhou, Xiangming Xiao, Stephen Grebby, Guoqing Zhang, Jinwei Dong

**Affiliations:** 1 School of Geographical Sciences, the University of Nottingham Ningbo China, Ningbo, 315100 China; 2 Key Laboratory of Land Surface Pattern and Simulation, Institute of Geographic Sciences and Natural Resources Research, Chinese Academy of Sciences, Beijing, 100101 China; 3 College of Geography and Environmental Science, Henan University, Kaifeng, 475004 China; 4 Department of Microbiology and Plant Biology, and Center for Spatial Analysis, University of Oklahoma, Norman, 73019 USA; 5 Nottingham Geospatial Institute, University of Nottingham, Nottingham, NG7 2TU UK; 6 Key Laboratory of Tibetan Environmental Changes and Land Surface Processes, Institute of Tibetan Plateau Research, Chinese Academy of Sciences, Beijing, 100101 China; 7 University of Chinese Academy of Sciences, Beijing, 100049 China

**Keywords:** Hydrology, Environmental impact

## Abstract

Lake systems on the Tibetan Plateau (TP) are important for the supply and storage of fresh water to billions of people. However, previous studies on the dynamics of these lakes focused on monitoring on multi-year scales and therefore lack sufficient temporal information. Here we present a new dataset comprising annual maps of big lakes (>10 km^2^) on the TP for 1991–2018, generated by utilizing all available Landsat images in conjunction with Google Earth Engine. The annual lake maps with high overall accuracy (~96%) highlight distinctive lake distribution and lake changes: (1) about 70% number and area of lakes concentrated in the Inner basin; (2) generally increasing trends in both the area (by 33%) and number (by 30%) of lakes from 1991 to 2018; (3) the total area changes were dominated by larger lakes (>50 km^2^) while more fluctuations in the lake number changes were found in medium lakes (10−50 km^2^). Our dataset infills temporal gaps in long-term inter-annual variations of big lakes, contributing towards enhanced knowledge of TP lake systems.

## Abstract (summary)

**Table:** 

Measurement(s)	lake area and number
Technology Type(s)	satellite images and coding
Sample Characteristic - Organism	lake
Sample Characteristic - Environment	plateau-climate
Sample Characteristic - Location	Tibetan Plateau

## Background & Summary

The Tibetan Plateau – often referred to as the Third Pole and ‘Water Tower of Asia’ – has the highest number and largest area of lakes (approximately 50,000 km^2^ or 50% of the total lake area) in China^1^. These lakes are sensitive to global and regional environmental changes, which makes them ideal indicators of climate change^2,3^. Monitoring lake area change is therefore essential with respect to regional environmental and climate change issues; it benefits an enhanced understanding of local cryosphere changes and their impact on ecosystems, energy and hydrological cycles, and on livelihoods^4,5^. However, most studies rely upon lake mapping results obtained for irregular or large time intervals (i.e., five-year interval) to analyze lake responses to climate change, and so a continuous annual history of detailed lake variations across the whole TP has not yet been examined^6–8^.

Most existing lake extent maps were generated using satellite imagery acquired at discrete time points, which limits their inter-year comparability since lakes undergo areal changes every year and even every day^1,3,9–18^. Some studies have also only focused on a specific lake type or area of the TP^1,6,19–23^. There are only a few datasets providing continuous annual lake maps^2,24^, however, they either do not provide lake maps with high spatial and temporal resolution over the TP since they address global- or national-scale issues, or they contain a relatively large degree of uncertainty due to problematic data or limitations in the suitability of the methods employed to map lakes on the TP^2^. For instance, the use of top-of-atmosphere reflectance data without atmospheric correction would likely introduce uncertainty into the global surface water body dataset generated by Pekel *et al*., since atmospheric correction is a necessary step to extract quantitative information from the satellite imagery to remove the effects of aerosols, clouds and cloud shadows^2,25,26^. Moreover, the use of ancillary maps and data products in generating this global water dataset could also have introduced additional inaccuracies and inconsistencies to the resultant maps, since these were generated from different resources using different standards and mapping approaches and likely have varying degrees of accuracy.

The most recent annual maps of China’s surface water area and storage (1989–2016) have poor data quality for 1989 and 1990 in the TP area due to limited Landsat imagery and, without further removal of glaciers or rivers, the data on lakes smaller than 30 km^2^ on the TP carries large uncertainties^27^. Therefore, it is challenging to assess the lake dynamics on the TP using any of the existing datasets. Consequently, the incomplete and non-continuous historic lake dynamics record hampers attempts to fully understand the underlying driving hydrologic mechanisms on the TP. To help overcome this, a more detailed and complete historic record of changes in the number and size of lakes on the TP is required.

This study presents a new continuous dataset of annual lake maps on the TP for 1991–2018, which is generated by using all of the available archived Landsat imagery in conjunction with the Google Earth Engine (GEE) cloud-computing platform. The dataset focuses on lakes >10 km^2^ in size (hereinafter termed ‘big lakes’) as they account for more than 90% of the total lake area on the TP^9,17^. Given the dominance of big lakes and the challenges in identifying smaller lakes in high mountain regions in satellite imagery (e.g., insufficient spatial resolution, obscuring effects of clouds, snow and topographic shadow), most studies – including the present one – utilize only lakes larger than 10 km^2^ to capture a reliable representation of lake change dynamics across the TP^6,8,28^. To achieve this, we apply an automatic mapping approach to detect water bodies on the TP that involves the use of spectral indices and the concept of water frequency to generate annual maps of the big lake extents. The result is a detailed annual lake record for the three most recent decades, which can provide an enhanced understanding of lake change dynamics across the TP and help establish the underlying driving mechanisms.

## Methods

### Study area

**Fig. 1:**
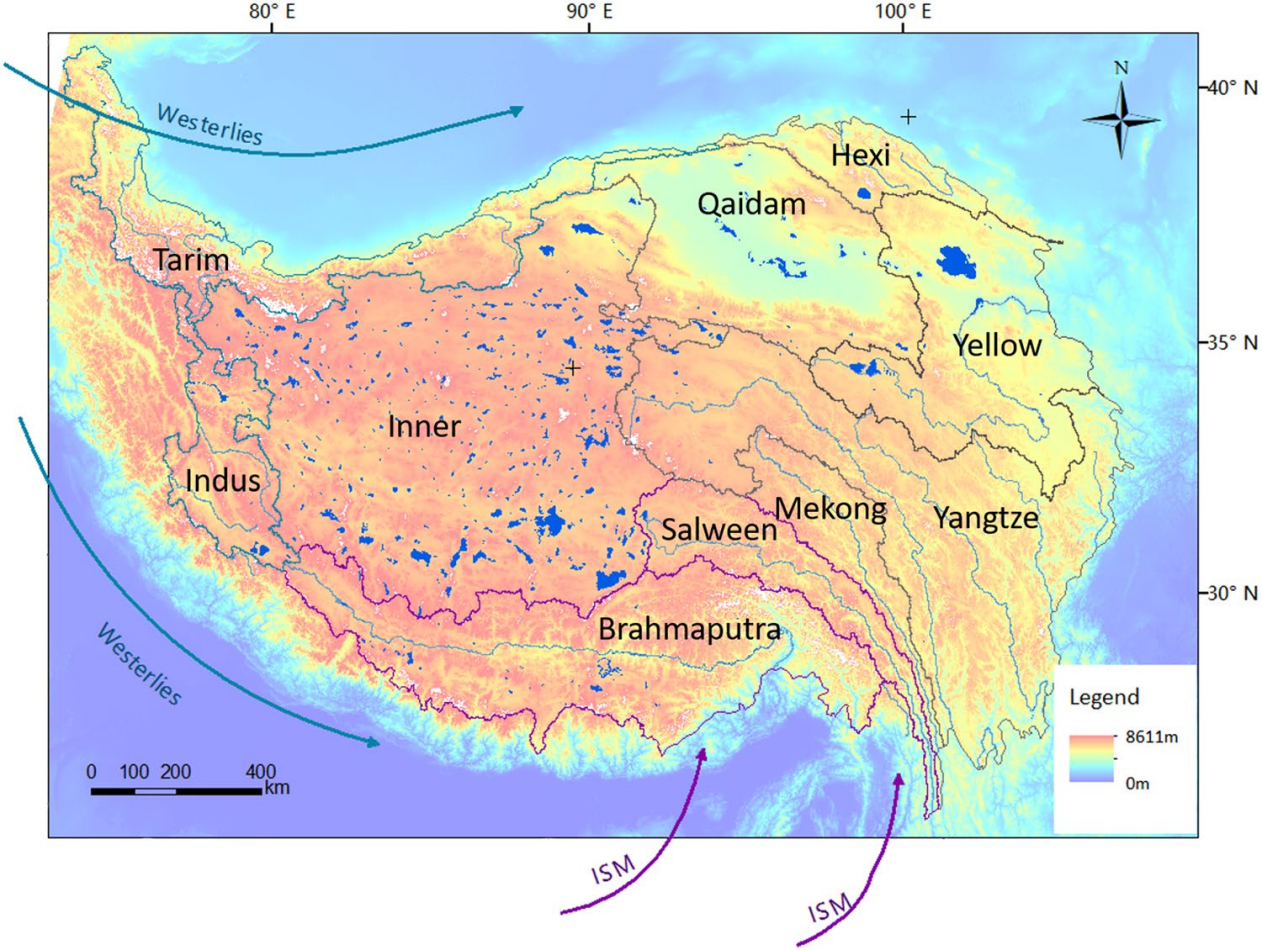
The Tibetan Plateau and ten main basins with hydrological features. Basin names are shown along with lakes in 2018 (dark blue polygons), China’s first order rivers on the TP (in light blue), and Glacier Area Mapping for Discharge from the Asian Mountains^36^ (in white). Arrows represent Westerlies (in blue) and ISM (in purple)^47,48^.

The TP is situated in central Asia (25°59′ N–39°49′ N; 73°29′ E–104°40′ E) and is the most extensive and highest plateau in the world, with an average elevation of more than 4000 m above sea level (a.s.l.) and an area of 2.5 × 10^6^ km^2,29^. There were ~1400 lakes of >1 km^2^ in size on the plateau in 2018, and 453 of these were larger than 10 km^2,17^. The changing hydrology on the TP has exerted huge impacts on ecosystems and human society, since it is the original water source for many essential rivers in Asia, supplying water to ~22% of Earth’s population for agricultural, industrial, and domestic use^30^. The climate across the TP varies significantly owing to its heterogeneous geography with annual average temperature from −4.1 °C in the Inner basin to 1.7 °C in Brahmaputra basin^31^. With its high elevation and broad surface, the plateau also acts as a barrier for the westerlies and monsoon atmospheric circulation. The plateau lies in the arid portion of the monsoon region and experiences a gradient of decreasing precipitation from east to west of 335–430 mm/yr^32^. The monsoonal climate area (Brahmaputra and Salween basins outlined in purple in Fig. 1) experiences summer rains and winter droughts, whereas the westerlies-controlled area (Tarim and Indus basins outlined in blue in Fig. 1) is extremely arid in winter with relatively more precipitation in summer. The other basins receive influences from both the Indian summer monsoon (ISM) and westerlies to some extent^33^, as indicated in Fig. 1.

### Source data

We obtained Landsat Collection 1 Tier 1 surface reflectance satellite imagery for Landsat 5 Thematic Mapper I, Landsat 7 ETM + , and Landsat 8 Operational Land Imager (OLI)/TIRS from the United States Geological Survey (USGS). All data in the collection have been atmospherically and geometrically corrected, and cross-calibration between different sensors has been applied^34^. Lakes were mapped using all the available imagery acquired during the period 1991–2018. It was not possible to map lakes at high temporal resolution prior to 1991 because the coverage is incomplete for the study area^2,35^. A summary of the source imagery used is shown in Table 1. Modern glacier polygons derived from Glacier Area Mapping for Discharge from the Asian Mountains (GAMDAM)^36^ were also used to avoid the possibility of glaciers being incorrectly mapped as lakes.

**Table 1:** An overview of all satellite imagery used in this study.

Sensors	Period	Number of images	Bands used
Landsat 5	1991–2011	39,379	Blue, Green, Red, NIR, SWIR1
Landsat 7	1999–2018	43,820	Blue, Green, Red, NIR, SWIR1
Landsat 8	2013–2018	18,569	Blue, Green, Red, NIR, SWIR1

NIR: Near-infrared; SWIR: Shortwave infrared

The study area is divided into ten basins as shown in Fig. 1^1,37^, due to the vast extent of the TP and its spatial heterogeneity. The boundaries of the TP were taken from the Datasets of the Boundary and Area of the Tibetan Plateau (DBATP), which is a result of long-term fieldwork and was released and revised in 2014^29^.

### Annual lake map generation

**Fig. 2:**
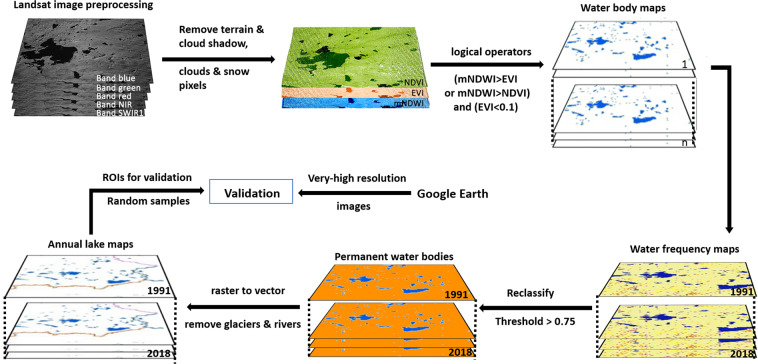
Methodological overview of continuous monitoring of lake dynamics on the TP using Landsat imagery.

An approach based on water frequency – which is the ratio of the number of water observations at each location to the total number of images used in a year^38^ – has proven effective for obtaining reliable representations of annual water bodies^27^. This is especially important for the TP, where significant seasonal variations in lake boundaries are observed^13,14^. The frequency map approach can also further help reduce the impact of cloud shadows and other cloud artifacts to improve the accuracy of the mapping. Also, this method can avoid the drawbacks of manual visual interpretation and mapping of lakes based on their appearance on remotely sensed imagery, which can be time-consuming over large areas and somewhat subjective. The mapping approach employed involves several steps, including image preprocessing, water body map generation, and annual lake map production based on the water frequency, as shown in Fig. 2.

#### Image preprocessing

All satellite images were processed for the removal of terrain and cloud shadow, clouds, and snow pixels using the CFMask tool designed for preparing Landsat imagery for accurate change detection^39^. Specifically, mountain shadows were masked using the function that calculates the solar azimuth and zenith angles using Landsat data and the 30 m Shuttle Radar Topography Mission (SRTM) digital elevation model. Clouds and their shadows were removed using the cloud and cloud shadow mask functions, respectively. Furthermore, the presence of permanent and temporary snow cover on the TP was masked using the corresponding snow mask function. This preprocessing resulted in the production of a collection of cloud-, shadow- and snow-free scenes of all the available Landsat imagery for the period, which comprised an average of 2839 images per year, ranging from 1927 images for the year 1991 to 4839 for 2018.

#### Generation of water body maps

Water body maps were subsequently generated from the preprocessed Landsat images using the Modified Normalized Difference Water Index (MNDWI), Normalized Difference Vegetation Index (NDVI), and Enhanced Vegetation Index (EVI), as reported in previous studies^25,38^:

1 $$MNDWI=\frac{{{\rm{\rho }}}_{{\rm{Green}}}-{{\rm{\rho }}}_{{\rm{SWIR}}1}}{{{\rm{\rho }}}_{{\rm{Green}}}+{{\rm{\rho }}}_{{\rm{SWIR}}1}}$$

2 $$NDVI=\frac{{{\rm{\rho }}}_{{\rm{NIR}}}-{{\rm{\rho }}}_{{\rm{Red}}}}{{{\rm{\rho }}}_{{\rm{NIR}}}+{{\rm{\rho }}}_{{\rm{Red}}}}$$

3 $$EVI=\frac{{{\rm{\rho }}}_{{\rm{NIR}}}-{{\rm{\rho }}}_{{\rm{Red}}}}{1.0+{{\rm{\rho }}}_{{\rm{NIR}}}+6.0{{\rm{\rho }}}_{{\rm{Red}}}+7.5{{\rm{\rho }}}_{{\rm{Blue}}}}$$

where *ρ*_*Blue*_, *ρ*_*Green*_, *ρ*_*Red*_, *ρ*_*NIR*_ and *ρ*_*SWIR1*_ are the surface reflectance values for the blue, green, red, near-infrared (NIR) and shortwave infrared-1 (SWIR1) bands of the Landsat sensors^40–42^.

The MNDWI enhances water features in the imagery and has been widely applied to identify the presence of water bodies in various regions^40–42^. It also has the added benefit of eliminating any residual effects associated with mountain shadows and light cloud cover^40–42^. Since low-lying vegetation in proximity to wet surfaces is one of the major causes of commission error in open surface water body mapping, in this study we combined MNDWI and vegetation indices (NDVI and EVI) using logical operators to improve the discrimination capabilities of the water body mapping algorithm^25,38^:

4 $$Water\,body=\left(MNDWI > EVIorMNDWI > NDVI\right)and\left(EVI < 0.1\right)$$

where ‘*MNDWI > EVI or MNDWI > NDVI*’ identifies pixels that have a stronger water signal than vegetation signal^25,38^, while ‘*EVI < 0.1*’ ensures that all vegetation pixels or mixed water-vegetation pixels can be removed^39^. Only pixels meeting the criteria were classified as corresponding to water bodies and all other pixels were classified as non-water pixels^25,38^. The criteria are effective in distinguishing water body from non-water pixels on Landsat images^27^, and so it was used to generate water body maps for each of the 101,768 individual Landsat images.

#### Generation of water frequency and annual lake maps

The individual water body maps for each year were collated and then used to compute yearly water frequency maps (28 in total). The water frequency for each pixel in a water frequency map was calculated according to Zou *et al*.^25^ as:

5 $$F\left(y\right)=\frac{1}{{N}_{y}}\mathop{\sum }\limits_{i=1}^{{N}_{y}}{W}_{y,i}\ast 100{\rm{ \% }}$$

where *F* is the water frequency,*y* is the year, *N*_*y*_ is the total number of Landsat observations for that pixel in that year, while *W*_*y,i*_ denotes whether a pixel in a water body map is classed as water (represented by a value of 1) or non-water (a value of 0).

A permanent water surface is underwater throughout the entire year^2^, which should correspond to an annual water frequency (*F*) of 100% in a frequency map. However, the water frequency of pixels corresponding to water bodies that do persist all year long can be less than 100% due to the obscuring effects of cloud cover and shadow in the satellite imagery. For instance, some clouds (i.e., optically thin clouds that were not masked) that obscure the presence of an actual permanent water body beneath have a chance of being classified as non-water in the water body maps, which in turn would reduce the water frequency of the corresponding pixels in the annual water frequency map. To overcome this problem, we set a threshold of *F* ≥ 75% to classify pixels in the frequency maps as permanent water pixels. The choice of the threshold value of 75% is in line with previous studies^25,27,38^ and has been confirmed that it was the most appropriate threshold value for indentifying permanent water bodies in the TP area. After applying the threshold to the water frequency maps, areas of permanent water were extracted using the Reclassify Tool in Arcpy as pixels comprising water for the vast majority of the year^38^. Only these permanent water bodies were selected for inclusion in the inter-annual dataset, since the temporary bodies of water could be associated with seasonal inundations and irrigation activities^2,41,43^.

Next, the permanent water body extents were converted from raster to vector format to produce annual lake maps. These water body polygons were then intersected with the modern glacier polygons to remove any glaciers that were incorrectly mapped as lakes. Furthermore, the presence of other water bodies, such as rivers, was manually detected and removed to further improve the quality of the lake mapping dataset. The remaining permanent water bodies were then considered to comprise only lakes. Finally, after filtering the dataset to retain the lakes with areas larger than 10 km^2^, the area and perimeter of each big lake were computed to facilitate the determination of inter-annual lake changes.

## Data Records

A total of 28 annual lake maps are provided for the entire TP for the period 1991–2018. The dataset is available at the figshare repository in the Esri shapefile format and a statistical summary file (10.6084/m9.figshare.13633880)^44^. The dataset is provided in the ESPG: 4326 (WGS_1984) spatial reference system. The 28 annual big lake maps comprise polygons demarcating the location and extent of each lake, and are attributed with information on the shape (Shape*), perimeter (Shape_Leng) and area (Shape_Area). These annual maps cover the geographic area from 73°29’ E to 104°40’ E longitude, and from 25°59’ N to 39°49’ N latitude. The maps can be visualized and analyzed in ArcGIS, QGIS or any similar software packages.

## Technical Validation

**Fig. 3:**
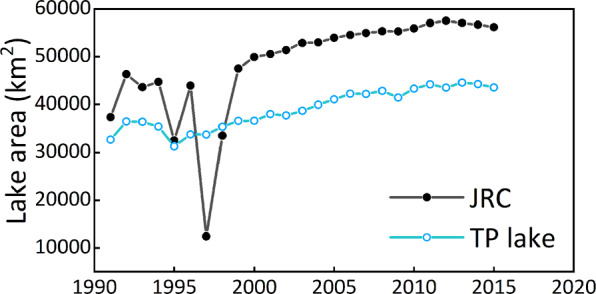
Comparision between JRC data and our results from 1991 to 2015.

In order to assess the thematic accuracy of the water body mapping algorithm, we chose to validate the accuracy of the 2018 lake map, given that this year had the highest temporal resolution of very-high resolution (VHR) images in Google Earth. In particular, the high number of VHR images acquired between June and October – when the presence of cloud cover is relatively high and lakes are close to their maximum area – increases the likelihood of obtaining cloud-free scenes that capture a representative perspective of the status of the lakes to permit a reliable validation of the mapping results^45^. A total of 1000 sample points inside the lake polygons and another 1000 samples outside the lake polygons were randomly generated across the TP using the ArcMap software. A validation dataset was then compiled by assigning each sample point as either permanent water or non-permanent-water through visual interpretation based on VHR images in Google Earth. Since the classification of 91 of the validation sample points was not possible due to persistent cloud or snow cover and other image limitations, the remaining 1909 sample points were used to calculate the accuracy through a confusion matrix. This was performed by comparing the designation of the validation points to those in the lake map to determine the proportion of points that were correctly classified. The results revealed an overall accuracy of 95.8% and a Kappa coefficient of 0.92 (Table 2). In addition, the user’s and producer’s accuracies of the lake maps are 92.1% and 98.9%, respectively. We also compared the dataset with another recent dataset of lakes larger than 1 km^2^ in Tibetan Plateau (V2.0) (mapped several specific years from 1970s to 2018)^1^, generated by using visual interpretation and NDWI. The area of lakes >1 km^2^ is only about 3.2–5.6% larger than the area of lakes >10 km^2^ mapped here, and that dataset also exhibits a similar change trend from 1991 to 2018 that further validates our method. The Joint Research Centre (JRC) dataset^2^, which represents significant progress in the remote sensing application of surface water mapping, was also used for comparision. Since the JRC dataset includes small lakes and streams, it has a moderately higher total lake area for TP region than that in our dataset. Nevertheless, the two datasets show very similar change trends on the whole (Fig. 3). The largest deviation between the two datasets occurs for 1997, when there is a substantial decrease of lake area in the JRC dataset and only a minor decrease was seen in our data (which has an overall accuracy of 93.4% for this year). The main differences between these two datasets could be due to subtle differences in the range of water bodies mapped (i.e., we excluded all rivers and lakes smaller than 10 km^2^, whilst the JRC dataset includes these), differences between using Lansdat top-of-atmosphere reflectance and surface reflectance data, as well as inherent differences between the mapping methods. Overall, the high mapping accuracy and comparison with the existing datasets attest the reliability and enhanced potential for ultising the 1991‒2018 annual lake map dataset for additional applications.

**Table 2:** Confusion matrix detailing the accuracy of the 2018 lake map (user’s accuracy refers to reliability and producer’s accuracy is the probability that a lake on the ground is classified as such).

	Lakes	Non-lakes	User’s Accuracy
Lakes	840	72	0.92
Non-lakes	9	988	0.99
Producer’s Accuracy	0.98	0.93	Overall accuracy = 95.8% Kappa coefficient = 0.92

Overall, accurate mapping of lakes depends on the quality and spatio-temporal resolution of the remotely sensed data and the choice of water body mapping algorithm^8,46^. In general, the uncertainty of the lake extent delineation using satellite data is inversely proportional to the spatial resolution of the images used, since the lakes or their associated boundary changes may be smaller in size than that of a pixel^8^. This is also the case for the identification of emerging and disappearing lakes. In this study, only the mapping of sizeable lakes (>10 km^2^) was performed, which is larger than the mapping unit (pixel size) of 30 m. While this excludes the analysis of smaller lakes, it is still possible to gain an accurate and reliable representation of lake dynamics across the TP given that 90% of the lakes are >10 km^2^. Furthermore, the ability to generate a consistent long-term dataset that captures the dynamics of the lakes is only possible due to the extensive archive of satellite imagery provided by the long-running Landsat earth observation program. Whilst satellites with improved spatial and spectral capabilities may facilitate more detailed mapping for the future and recent past, a long-term historical analysis of lakes on the TP is inherently constrained by the imaging capabilities of the Landsat satellites.

In this study, the use of an automatic method was critical for large-scale lake mapping over a long time period, as the manual interpretation of over 100,000 individual Landsat images would have been an extremely laborious task. Also, compared to other studies that use only single images to extract lakes for each year, calculating the water frequency helps to improve the mapping accuracy by mitigating the effect of clouds and their shadows, as well accounting for seasonal changes of the lakes^25^. This was achieved by applying a water body frequency threshold of 75% to capture only permanent lakes, therefore increasing the reliability of the observed inter-annual variation trends^2,38,46^. The water frequency approach also mitigates potential differences in the reliability when mapping using imagery acquired from Landsat 5, 7 and 8^46^, particularly those surrounding the number of observations available for each year based on the operational periods of the satellites. Although there were an average of 1790 good observations each year for 1991‒1998 from Landsat 5, 3981 images each year for 1999‒2012 acquired from Landsat 5 and 7, and 5286 satellite images each year for 2013‒2018 from Landsat 7 and 8, all map products performed comparably well (with accuracy >95%^2,27^) in capturing surface water regardless of the platform or number of observations. Therefore, the influence of using three Landsat sensors on the mapping results is considered negligible. Furthermore, a consistent method of lake mapping was applied to 28 years’ worth of data, which also helps to further reduce any uncertainties in the observed trends^38^. Finally, the intersection with glaciers and manual removal of rivers further improves the quality of the lake mapping results.

In the future, with improved access to higher spatial resolution data, the mapping of smaller lakes and the analysis of the drivers could be undertaken to analyze contemporary lake change dynamics. In addition, the method outlined here could be applied to imagery as it is acquired, in order to rapidly update the lake maps for the TP in near-real time. The ability to map and analyse seasonal (intra-year changes) is currently restricted by the lack of sufficient observations needed to reliably generate monthly or bi-monthly water frequency maps, due to the prevalent cloud cover over parts of the TP, especially during the summer. However, with access to satellites or constellations of satellites with a shorter revisit time in the future this will likely become possible as higher frequency acquisitions would maximise the opportunity to obtain more cloud-free images.

## Usage Notes

**Fig. 4:**
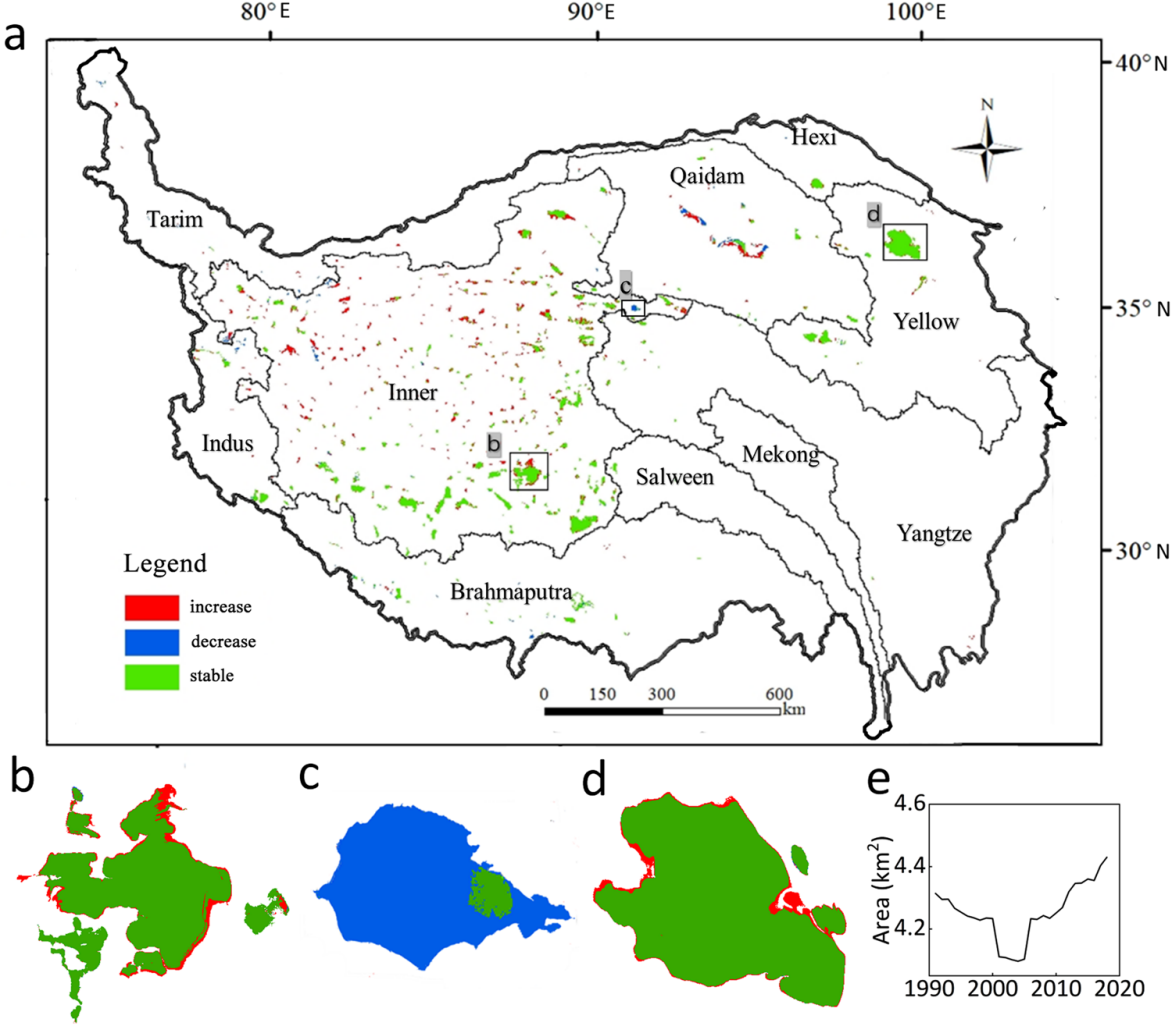
Lake area variation. (**a**) Lake size changes across the TP; (**b**) Siling Co and (**c**) Zhuo Nai Lake area variations; (**d**), (**e**) Qinghai Lake area variation from 1991 to 2018.

The lakes on the TP are highly dynamic and mapping their inter-annual variations can provide enhanced insights into the effects of climate oscillation or extreme climate events. Tracking the inter-annual lake dynamics can also provide a valuable indication of the future of the TP hydrological system. In this study, we have produced maps with relatively high temporal resolution (1 year) and spatial resolution (30 m) for a long time-series (28 years) for all TP lakes larger than 10 km^2^. This continuous long-term record therefore allow us to examine the lake changes in detail and ensures that any crucial and relatively rapid changes in the trends are not missed. The annual lake maps are valuable for investigating the spatial heterogeneity of lake change patterns across the plateau and for investigating the changes of specific lakes. Particularly, the high accuracy of the maps ensures the quality and reliability of the obtained change records for those important lakes (Fig. 4). If combined with climatic data of high spatial and temporal resolution or change records of environmental elements like glaciers, permafrost and vegetation, the annual lake maps would allow for an in-depth analysis of the mechanism behind the changes or the linkage between them. The lake boundaries are also important for the validation of global and regional hydrological modelling and can be used to improve the predication capability of models to aid a better understanding of the water resources, which would further inform policy-making and support sustainable development in the region. The data may also contribute to regional and local flood risk mapping across the TP.

### Data statistical properties

**Fig. 5:**
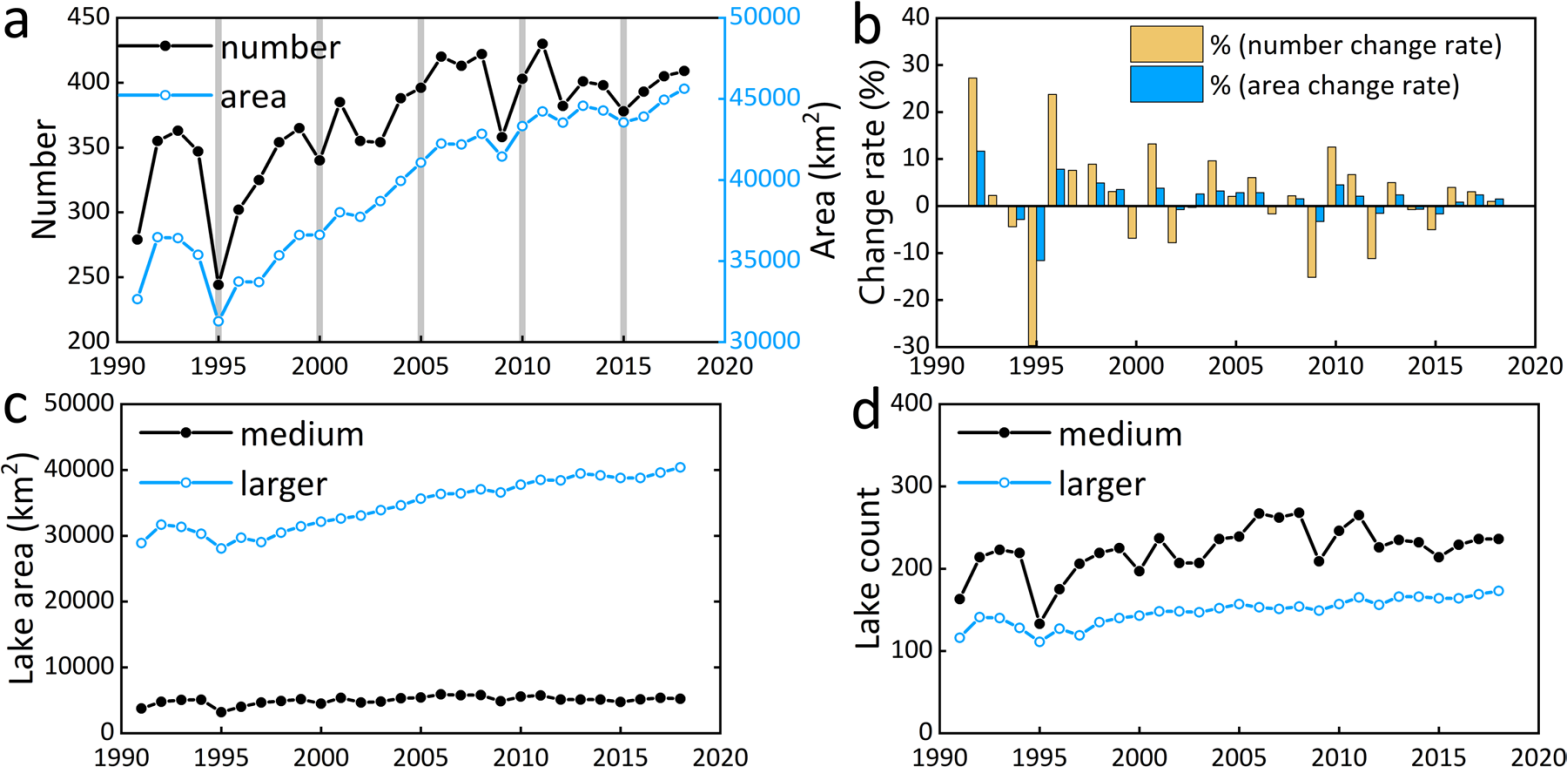
Lake area and number dynamics. (**a**) Annual total number and area of lakes larger than 10 km^2^ on the TP from 1991 to 2018 (grey columns indicate what data will a five-year-map of lakes on the TP look like); (**b**) Lake number and area change rates; (**c**) lake area and (**d**) number change for medium lakes (10−50 km^2^) and larger lakes (>50 km^2^), respectively.

We performed a statistical analysis on the dataset to further demonstrate the value of the new annual lake maps. With the presented continuous lake maps, we are able to track the changes associated with big lakes across the TP on an annual basis during 1991–2018. Overall, the TP lakes experienced a significant expansion from 1991 to 2018 (Fig. 5), with a considerable increase of 12976 km^2^ in area (40%) and 130 in number (47%)^44^. This corresponds to overall change rates of lake area and number of 463 km^2^/year and 4.6 lakes/year, respectively. In 2018, there were 409 lakes >10 km^2^ on the TP, comprising a total area of 45621 km^2^. In terms of the dynamics, the annual lake change record shows an increase in area from 1991 to 1992, followed by a sharp decrease in 1995 and then a significant and continuous increase since 1995, except in 2009 and 2015 when decreases occurred (Fig. 5a,b). These fluctuations in lake area observed in 1992, 2009 and 2012 would not be detectable in five-year interval datasets, which were the highest temporal resolution lake datasets covering the TP prior to this study (indicated in Fig. 5a). The change in the number of lakes follows a very similar pattern to the lake area (Fig. 5a,b). Similarly, a five-year interval lake number dataset with record of only 1995, 2000, 2005, 2010 and 2015 would lack information for lake dynamics within those five-year intervals so that fewer fluctuations will be seen (Fig. 5a). Overall, the inter-annual fluctuation in lake area and number is highest during 1991–1996 and around 2010 (Fig. 5b). Larger lakes (>50 km^2^) are seen to have contributed to the overall change in lake area across the TP (Fig. 5c). From 1991 to 2018, the total area of the larger lakes increased considerably by 11521 km^2^ (39.9%), while the medium-sized lakes (10−50 km^2^) experienced a smaller areal increase of 1455 km^2^ (38.7%). Although both the numbers of larger (from 116 to 173) and medium (from 163 to 236) lakes significantly increased during 1991–2018 (Fig. 5d), the fluctuation in the number of medium lakes is greater than that of larger lakes, most notably in 1995 and 2010.

**Fig. 6:**
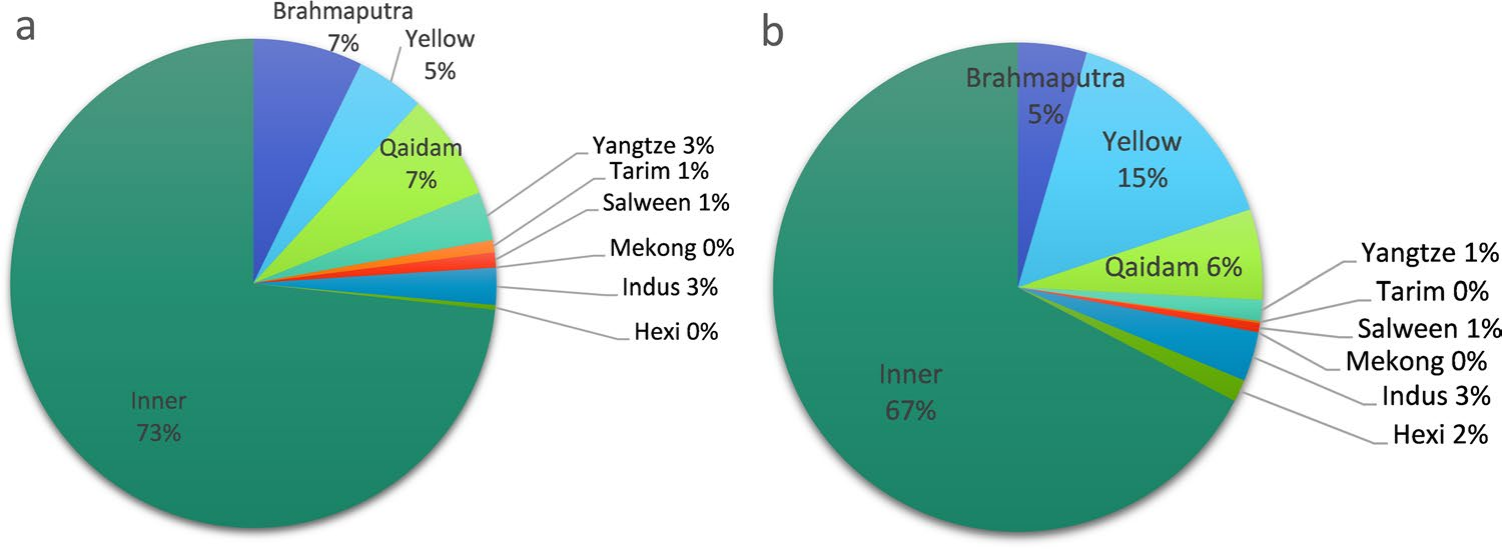
Distribution of lakes on the TP. Lake number (**a**) and area (**b**) averaged for 1991–2018.

We further calculated both the number and area of lakes for each basin and found that the lakes are unevenly distributed across the TP (Fig. 6). The Inner basin was found to have the highest average proportion of lakes with 73% of the total, followed by the Qaidam basin and Brahmaputra basin (Fig. 6a). On average, there are only 0 to 15 lakes located in Tarim basin, Yangtze River basin and Salween basin, while the Mekong basin does not contain any lakes larger than 10 km^2^. The Inner basin has the largest lake area (67%), followed by Yellow (15%) and Qaidam (6%). Salween, Yangtze, Mekong and Tarim basins have the smallest total lake area on the TP.

## Data Availability

The annual maps of lakes larger than 10 km^2^ from 1991 to 2018 were produced using GEE platform. Key JavaScript code developed for this work are openly shared with the scientific community at figshare repository^44^. GEE should be used to access and edit the code.
